# Perioperative Spinal Cord Injury in the Setting of Whipple Procedure for Pancreatic Adenocarcinoma in a Patient With a History of Severe Cervical Stenosis

**DOI:** 10.7759/cureus.65412

**Published:** 2024-07-26

**Authors:** Benjamin Freedman, Christopher M Russo, Nicole Batt, Mitchell Harrison, Davis Frease

**Affiliations:** 1 School of Medicine, Uniformed Services University of the Health Sciences, Bethesda, USA; 2 Anesthesiology, Walter Reed National Military Medical Center, Bethesda, USA

**Keywords:** physical medicine and rehabilitation, anesthesiology, neurosurgery, posci, traumatic spinal cord injuries, whipple procedure, american spinal injury association, chronic spinal canal stenosis, pancreaticoduodenectomy, perioperative spinal cord injury

## Abstract

Perioperative spinal cord injury (POSCI) is a form of traumatic acute spinal cord injury (TSCI) in the perioperative setting that is a rare but feared complication associated with severe morbidity and mortality, often resulting in significant functional impairment and significant healthcare costs for the patient. Here, we present a case report of a 65-year-old male with a past medical history of hypertension (HTN), type-2 diabetes mellitus (T2DM), stage 4 chronic kidney disease (CKD4) with a one-year history of anorexia, weight loss, jaundice, and right lower quadrant (RLQ) pain. He underwent an endoscopic ultrasound, which showed pancreatic atrophy, marked dilation of the main pancreatic duct, and a poorly defined pancreatic head mass. The patient underwent a successful pancreaticoduodenectomy and was extubated in the operating room and transferred to the surgical intensive care unit (SICU) on an oxygen face mask without complication. Four hours later it was noted that the patient’s neurological exam had acutely changed with loss of motor and sensory function from the C7 dermatome down. The patient remained stable from a cardiopulmonary standpoint, and he was urgently transferred for emergency imaging of his brain and spinal cord, which demonstrated evidence of chronic spinal canal stenosis, complete cord flattening at the C5 level with profound cord edema centered at the C5 level extending from C3 to T1. Following diagnosis, neurosurgery was consulted at the SICU and a comprehensive neurological exam was performed. It was determined the patient had a grade A injury via the American Spinal Injury Association (ASIA) Impairment Scale and required an emergency cervical laminectomy. The patient was taken back to the operating room (OR) and an open cervical laminectomy was performed from C3 to C7 without any intraoperative complications. The patient was managed by a multidisciplinary SICU team for both his pancreaticoduodenectomy, perioperative traumatic acute spinal cord injury, and subsequent multilevel cervical laminectomies. The patient had a purposeful neurological recovery over the following weeks and was ultimately discharged to an inpatient physical rehabilitation facility.

## Introduction

Perioperative spinal cord injury (POSCI) is a rare but catastrophic perioperative complication associated with significant morbidity and mortality. It is more commonly seen in patients with traumatic and unstable cervical spine injuries or underlying preexisting chronic pathologies predisposing them to increased susceptibility to complications involving the spinal cord [1,2]. Here, we report a case of a 65-year-old male patient with recently diagnosed pancreatic head cancer, hypertension (HTN), type-2 diabetes mellitus (T2DM), and stage 4 chronic kidney disease (CKD4) who underwent a successful pancreaticoduodenectomy (Whipple procedure). His perioperative course was complicated by an acute change in neurological exam four hours following his surgery concerning for perioperative cervical spine injury of undetermined etiology. Upon identification of the change in neurological exam, emergency diagnostic imaging of his brain and spinal cord was obtained and demonstrated evidence of extensive chronic spinal canal stenosis with acute complete cord flattening at the C5 level with profound cord edema centered at the C5 level and extending from C3 to T1. Neurosurgery was urgently consulted for evaluation and subsequently performed an emergency multilevel cervical laminectomy.

## Case presentation

**Figure 1:**
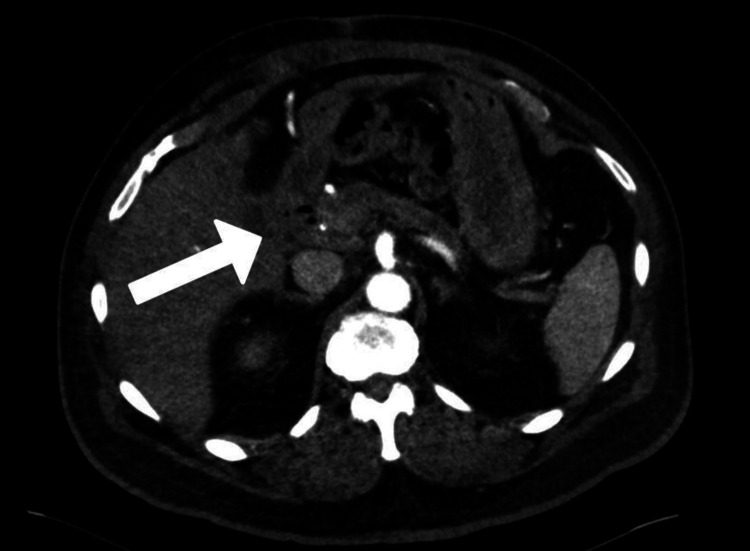
CT pancreas depicting pancreatic head mass (arrow) with marked dilation of the main pancreatic duct and pancreatic tail atrophy. CT: Computed tomography

A 65-year-old male with a past medical history of HTN, T2DM, and CKD4 with a one-year history of anorexia, weight loss, and acute chronic right lower quadrant (RLQ) pain presented to the emergency department (ED), and following physical examination, basic diagnostic labs, and pain control rapidly underwent a right upper quadrant (RUQ) ultrasound that showed cholelithiasis. However, the study was deemed technically challenging and of questionable diagnostic quality due to the patient's pain. Following the RUQ ultrasound, a computed tomography (CT) study of his abdomen and pelvis without contrast was obtained and notable for evidence of cholelithiasis and mucosal thickening of the gallbladder wall to 5.6 mm with significant pericholecystic fat stranding. Additionally, the patient's common bile duct and main pancreatic duct were dilated to 8 mm and 5.4 mm, respectively, with evidence of moderate to severe inflammatory changes localized to the pancreatic head. General surgery was consulted and admitted the patient for observation, pain control, and magnetic resonance cholangiopancreatography (MRCP) the following morning in an effort to further delineate if the patient was suffering from cholecystitis or choledocholithiasis due to the presence of ductal dilation. The patient was admitted in a hemodynamically stable condition and underwent the MRCP, which favored cholecystitis. However, in addition, pancreatic atrophy was noted with marked dilation of the pancreatic duct and an abrupt cut-off in the region of the pancreatic head most concerning for a neoplastic mass, which recommended further evaluation via endoscopic ultrasound (EUS). An EUS was performed by interventional gastroenterology, and a hypoechoic mass in the pancreatic head mass measuring 28 mm by 29 mm was identified and sampled via EUS-guided fine needle aspiration (FNA), which showed atypical cells exhibiting crowding, disorganization, and nuclear pleomorphism concerning for pancreatic malignancy. These findings resulted in urgent surgical consultation in conjunction with a CT pancreas protocol (see Figure 1 below) to further characterize the mass, and evaluate the surrounding vascular structures for surgical planning. Of note, the CT pancreas showed the mass was periampullary, which is a subgroup of tumors arising within 2 cm of the major duodenal papilla (ampulla of Vater) in addition to significantly enlarged paraaortic and retrocaval lymph nodes all greater than 2 cm. It was at this time the patient was enrolled in our institution’s tumor board for further discussion by a multidisciplinary team of specialists based on the patient’s history, comorbidities, and tumor imaging characteristics. In addition to tumor board evaluation, he was also evaluated by the anesthesiology, cardiology, pulmonology, and endocrinology services based on his multiple medical comorbidities in anticipation for possible urgent and time-sensitive oncologic surgery. While undergoing extensive diagnostic work-up, it was noted that the patient had significantly elevated serum tumor markers with a cancer embryonic antigen (CEA) level of 11.5 ng/mL (normal range: 0.0-4.7 ng/mL) and a carbohydrate antigen (CA 19-9) level of 357 U/mL (normal range: 0-35 U/mL) which are associated with worse perioperative outcomes and survival. Following a comprehensive discussion and thorough evaluation by the multidisciplinary tumor board team, the final recommendation was to pursue a pancreaticoduodenectomy with the surgical oncology service.

During his paranesthesia evaluation, he reported no personal or family history of complications of anesthesia, no known drug allergies to food or medications, and adherence to presurgical noting by mouth guidelines. The patient was transported to the operating room (OR) and adequately preoxygenated with 100% O_2_ for 10 minutes and standard American Society of Anesthesiology (ASA) monitors were applied. Upon the patient reaching end-expiratory oxygen saturation of (EtO_2_) of 85% general anesthesia was induced with lidocaine, propofol, fentanyl, and rocuronium without any significant hemodynamic or cardiopulmonary complications. With his head and neck in a neutral position, direct laryngoscopy revealed a grade 1 view of the vocal cords per the Cormack-Lehane Grading System and tracheal intubation was successful with size 8.0 endotracheal tube (ETT) requiring only one attempt. A postinduction radial arterial line was then placed without complication. Maintenance of anesthesia was managed with sevoflurane at 1.0 mean alveolar concentration (MAC) throughout the duration of the case. The pancreaticoduodenectomy was completed without any notable intraoperative complications or the necessity for vasoactive medication or blood product transfusion. The patient was successfully weaned from the ventilator, extubated safely to an oxygen mask in the OR, and transported with continuous monitoring to the surgical intensive care unit (SICU) in stable condition.

**Figure 2:**
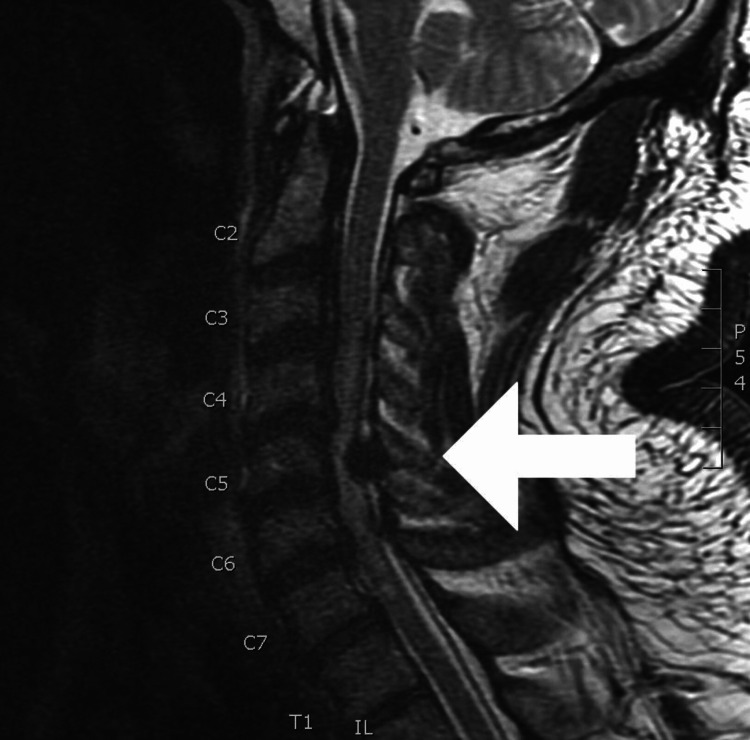
T2-weighted cervical spine MRI exhibiting evidence of chronic spinal canal stenosis with complete cord flattening at the C5 level with profound cord edema centered at the C5 level extending from C3 to T1. MRI: Magnetic resonance imaging

In the immediate postoperative phase, the patient denied nausea and pain, and his neurological examination was at his preoperative baseline. Roughly four hours later while the surgical team was performing routine postoperative wound checks and dressing changes with the SICU nursing staff, it was noted that the patient’s neurological exam had acutely changed with complete loss of motor and sensory function from the C7 dermatome down. The patient’s vital signs remained stable at this time without any indication of cardiopulmonary compromise. Based on his acute change in the neurological exam, emergent imaging of his brain and spinal cord was obtained with a demonstration of chronic spinal canal stenosis, complete cord flattening at the C5 level with profound cord edema centered at the C5 level extending from C3 to T1 (see Figure 2 below).

Neurosurgery was immediately consulted on the acute change in neurologic exam and imaging findings and following their rapid evaluation it was determined that the patient had suffered a grade A spinal injury per the American Spinal Injury Association (ASIA) Impairment Scale, and he was urgently transported back to the OR for an open cervical laminectomy from C3 to C7 that was completed without any intraoperative or postoperative complication [2]. The patient was managed by a multidisciplinary SICU team for both his pancreaticoduodenectomy, perioperative traumatic acute spinal cord injury, and subsequent multilevel cervical laminectomies. He had a purposeful neurological recovery over the following weeks gaining meaningful function back in his upper extremities and was ultimately discharged to an inpatient physical rehabilitation facility.

## Discussion

The spinal column contains a significant amount of neurological, vascular, and musculoskeletal anatomy condensed into a relatively small area with an estimated average adult cross-sectional area of approximately 80 mm. It is further surrounded by additional supportive structures (muscle, cartilage, fascia) and intricate neurovascular anatomy [1,2]. In the average adult, the vertebral column consists of 33 vertebrae (seven cervical, 12 thoracic, five lumbar, five sacral, and four coccygeal) all containing the spinal canal through which the spinal cord and supplying vasculature travel from the brain to its termination point at the conus medullaris and ultimately the filum terminale externum within the fused vertebrae of the coccyx. A single anterior spinal artery and two posterior spinal arteries account for most of the blood supply to the spinal cord. Both the anterior and posterior spinal arteries are derived from the segmental arteries connecting to the aorta and are essential for its continued oxygenation and tissue perfusion. A comprehensive understanding of the spinal cord anatomy and its intimately related structures is essential to understanding the pathophysiology, prevention, and perioperative management of perioperative acute spinal cord injury.

Traumatic spinal cord injuries can be graded from A to E according to the ASIA Impairment Scale. Grade A consists of complete spinal cord injury with no preservation of function below the level of injury, grade E consists of normal motor and sensory function, and grades B-D are varying levels of incomplete neurological function [3]. POSCI is a rare, but feared, complication of spine surgery and has an estimated incidence of 0-3% depending on existing chronic pathology, mechanism of traumatic injury (if applicable), intraoperative complications, spinal cord level, and surgical approach [2,3]. The underlying pathology of POSCI can be further broken down into primary and secondary insults. The primary mechanism of injury is typically defined as direct central neuronal tissue injury and can include isolated or a combination of compression, impaction, laceration, shearing, distraction, or ischemia [2,3]. Major contributing factors to the primary injury perioperatively typically involve an underlying pathological process such as developmental stenosis, preexisting myelopathy, congenital spinal deformities, osteopenia, and any chronic conditions that result in erosive synovitis to the ligaments of the cervical spine, most commonly rheumatoid arthritis [3].

The preexisting history of chronic spinal cord stenosis in the described patient likely played a significant role in the unanticipated and unexpected outcome experienced. This highlights the importance of constant consideration of POSCI by surgeons, anesthesiologists, and OR staff regardless of the type of case to include non-spine related cases in patients with a significant preexisting pathology affecting the spinal cord. This point is highlighted in this case as this patient was undergoing a pancreaticoduodenectomy (Whipple procedure) but developed POSCI. Risk identification and stratification are paramount in these high-risk surgical patients. When underlying spinal pathology is identified, such patients should have a comprehensive evaluation by a physician and their condition should be adequality optimized prior to undergoing surgery (if elective).

Once an injury to the spinal cord occurs in the perioperative setting, early detection and identification are key and are the most important determinants for the patient’s outcome, residual disease burden, postdischarge functionality, and quality of life moving forward. It is unknown at what point in the perioperative setting this patient suffered his complication. Nevertheless, such injuries are most often a result of excessive neck extension during laryngoscopy and/or intubation and following induction of general anesthesia the manual positioning of the patient by the anesthesia and surgical teams. Following the initial insult, regardless of its etiology, disruption of blood flow/oxygen delivery to the neuronal tissue can result in ischemia. If this process is not rapidly identified and corrected and neuronal tissue perfusion is not restored, irreversible damage to the neuronal tissue may occur [2,3]. Neuronal tissue ischemia results in the rapid release of cytokines and inflammatory and vasoactive proteins, resulting in inflammation that further exacerbates the already impaired oxygenation of the spinal cord [2,3]. An additional consideration in cases of POSCI is avoiding hemodynamic instability resulting from reduced blood volume (hemorrhage, anemia, hypovolemia) and the use of vasopressors, both of which can further worsen tissue perfusion and oxygenation to the acutely injured neuronal tissue.

This patient’s spinal injury was determined to be grade A, warranting urgent surgical intervention to prevent additional ischemic injury and permanent long-term sequelae. Additional medical management for an injury above T6 includes hemodynamic support specifically monitoring for hypotension, cardiac arrhythmia, and respiratory distress [3]. Increasing a patient's hemoglobin levels and utilization of vasopressors may be necessary to maintain a mean arterial pressure (MAP) above 85-90 mmHg and enhance spinal perfusion and potential neurologic recovery [2,3]. There have also been reports of hyperbaric oxygen therapy assisting in the neurologic recovery of patients with spinal cord ischemia by increasing available oxygen for tissue delivery in the blood [4]. After spinal cord injury has been identified which requires surgical intervention, maintaining a MAP above 70 appears to be associated with postoperative motor score improvement [4,5]. Additionally, evidence points to improved neurological outcomes with decompression of the spinal cord injury within 24 hours of the inciting event [5,6]. The early recognition and urgent surgical intervention with this patient likely contributed to his meaningful recovery over the following weeks postsurgery.

## Conclusions

POSCI is a rare but feared complication that can occur in the perioperative setting. While it is more commonly associated with head, neck, and spine surgery, it can occur in any surgical or procedural case. The risk of POSCI is particularly increased in patients with underlying comorbidities affecting the spinal cord and its supporting structures such as spinal stenosis, preexisting myelopathies, congenital spinal deformities, osteopenia, and rheumatoid arthritis due to the erosive synovitis of the ligaments of the cervical spine. All members of the operating room team (surgeons, anesthesiologists, nurses, technicians, etc.) should be cognizant of preexisting pathology or surgical procedures that put their perioperative patients at a heightened risk of developing POSCI. Lastly, increased caution and safety should be practiced by all members of the operating room team when manipulating and/or moving patients while under general endotracheal anesthesia (GETA). 
